# Silicon Oxycarbide—Tin Nanocomposite as a High‐Power‐Density Anode for Li‐Ion Batteries

**DOI:** 10.1002/advs.201901220

**Published:** 2019-07-28

**Authors:** Romain J.‐C. Dubey, Pradeep Vallachira Warriam Sasikumar, Frank Krumeich, Gurdial Blugan, Jakob Kuebler, Kostiantyn V. Kravchyk, Thomas Graule, Maksym V. Kovalenko

**Affiliations:** ^1^ Laboratory of Inorganic Chemistry Department of Chemistry and Applied Biosciences ETH Zürich CH‐8093 Zürich Switzerland; ^2^ Laboratory for Thin Films and Photovoltaics Empa Swiss Federal Laboratories for Materials Science & Technology CH‐8600 Dübendorf Switzerland; ^3^ Laboratory for High‐Performance Ceramics Empa Swiss Federal Laboratories for Materials Science & Technology CH‐8600 Dübendorf Switzerland

**Keywords:** electrochemical energy storage, lithium, nanocomposites, silicon oxycarbide, tin metal

## Abstract

Tin‐based materials are an emerging class of Li‐ion anodes with considerable potential for use in high‐energy‐density Li‐ion batteries. However, the long‐lasting electrochemical performance of Sn remains a formidable challenge due to the large volume changes occurring upon its lithiation. The prevailing approaches toward stabilization of such electrodes involve embedding Sn in the form of nanoparticles (NPs) in an active/inactive matrix. The matrix helps to buffer the volume changes of Sn, impart better electronic connectivity and prevent particle aggregation upon lithiation/delithiation. Herein, facile synthesis of Sn NPs embedded in a SiOC matrix via the pyrolysis of a preceramic polymer as a single‐source precursor is reported. This polymer contains Sn 2‐ethyl‐hexanoate (Sn(Oct)_2_) and poly(methylhydrosiloxane) as sources of Sn and Si, respectively. Upon functionalization with apolar divinyl benzene sidechains, the polymer is rendered compatible with Sn(Oct)_2_. This approach yields a homogeneous dispersion of Sn NPs in a SiOC matrix with sizes on the order of 5–30 nm. Anodes of the SiOC/Sn nanocomposite demonstrate high capacities of 644 and 553 mAh g^−1^ at current densities of 74.4 and 2232 mA g^−1^ (C/5 and 6C rates for graphite), respectively, and show superior rate capability with only 14% capacity decay at high currents.

## 1 Introduction

At present, the replacement of graphite—the conventional anode material for Li‐ion batteries (LiBs)—is a key to improving the energy density of present‐day LiBs. Among possible alternatives, tin had long been thought to be a compelling alternative anode material that exhibits a high theoretical Li‐ion storage capacity of 994 mAh g^−1^ (7246 mAh cm^−3^) for Li_22_Sn_5_ alloy formation and a low average delithiation potential of 0.6 V versus Li^+^/Li.^1^ The stable electrochemical performance of Sn, however, remains a notoriously challenging issue, mainly because of large volume changes of up to 260% upon lithiation, resulting in pulverization and loss of electrical percolation within the electrode.^2^ Additionally, the rupture and reformation of the solid‐electrolyte interface (SEI) layer caused by these large volume changes result in continuous electrochemical decomposition of the electrolyte. Overall, any practical strategy to mitigate this issue should utilize not only nanostructuring of Sn but also effective disentanglement of alloying and SEI‐related processes. First, reducing the size of Sn to under 100 nm prevents particle fragmentation.^3^ At the same time, a suitable matrix is then needed to prevent the agglomeration of Sn particles. With regard to their ability to store Li ions, diverse matrices have been tested and can be categorized as electrochemically inactive (e.g., Sn metal oxides,^4^ SnFe_3_C,^5^ CoSn_2_O*_x_* 6 and FeSn_2_/CoSn_2_;^7^ at least one electronically conductive component does not store Li ions and hence acts as a matrix) or active (GeSn^8^ and SbSn;^9^ all elements alloying with Li) and those showing rather limited faradaic and capacitive storage of Li ions, such as amorphous and lightweight carbon‐based composites.^10^ The active matrix approach has been attractive due to high theoretical charge storage capacities but has thus far been ineffective for the overall stabilization of Sn‐containing electrodes due to relatively large volume expansion of active components upon lithiation. Inactive carbon‐based matrices, on the contrary, allow for long cycling stability, effectively improving the electrical contact to Sn active materials and hindering their shedding and aggregation. Yet this comes at the expense of the overall charge storage capacity. The quest for suitable matrix engineering therefore continues. It has become apparent that the desired attributes of both extremes—high Li‐ion storage, low volume expansion upon lithiation and sufficient electronic conductivity—need to be harnessed in a multicomponent, synergistic architecture.

In this study, polymer‐derived ceramics, namely, silicon oxycarbide (SiOC for simplicity), were chosen as appealing candidates for stabilizing Sn inclusions. This matrix features a high Li‐ion storage capacity ranging from 600 to 700 mAh g^−1^, low volume expansion upon lithiation of about 7%^11^ and high electronic conductivity.^12^ The SiOC microstructure consists of tetrahedral SiOC units along with a segregated free carbon (C_free_) phase.^13^ It had been found that a high level of carbon disorder in C_free_ phase yields a higher Li‐ion storage capacity of SiOC.^14^ The synthesis of a SiOC/Sn nanocomposite via a PDC route was first demonstrated by Kaspar et al.^15^ and includes mixing a polysilsesquioxane, a polysiloxane and Sn acetate (Sn(Ac)_2_) and is followed by pyrolysis at 1000 °С. However, due to the inhomogeneous distribution of the components caused by the incompatible polarities of the preceramic polymer network with Sn(Ac)_2_, the obtained spherical Sn nanoparticles (10–45 nm) were not uniformly distributed within the SiOC matrix, thus leading to poor rate capabilities. Moreover, SiOC matrix comprised a highly ordered C_free_ phase that significantly reduced its Li‐ion charge storage capacity.^16^

Inspired by the work of Kaspar et al.,^15^ we report a novel synthetic route yielding homogeneously distributed Sn within a SiOC matrix. First, we altered the polarity of the sidechains of the polysiloxane precursor through a solvent‐free hydrosilylation reaction. Second, we used Sn ethylhexanoate (Sn(Oct)_2_) as a stable, inexpensive, and nontoxic Sn precursor. Contrary to Sn(Ac)_2_, the counterion in Sn(Oct)_2_ has a long hydrocarbon tail, which decreases its polarity. The complete miscibility of Sn(Oct)_2_ with the liquid polysiloxane precursors and the absence of a reaction with the sidechains of polysiloxane were paramount for obtaining a homogeneous dispersion of Sn in the SiOC matrix. Importantly, the synthesized SiOC matrix was composed of highly disordered C_free,_ yielding high Li‐ion storage capacities of ≈600 mAh g^−1^ measured in the voltage range of 5 mV–1.5 V versus Li^+^/Li. We determined that the synthesized SiOC/Sn nanocomposites exhibit a high rate capability as an anode material in Li‐ion batteries, delivering a high capacity of 553 mAh g^‐1^ at a current density of 2232 mA g^‐1^ (≈6C for a graphite anode).

## 2 Results and Discussion

### 2.1 Synthesis and Characterization of the SiOC/Sn Nanocomposites

**Figure 1:**
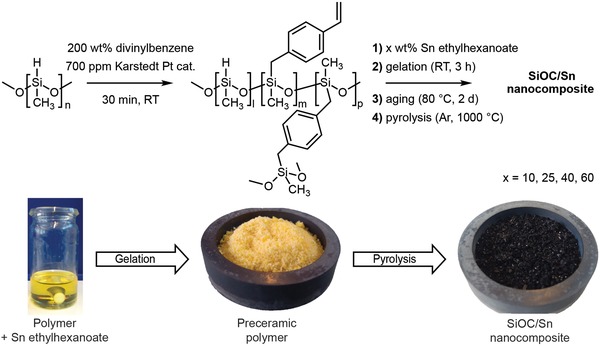
Schematics of the synthetic procedure of SiOC/Sn nanocomposites and photographs of the precursor solution of Sn(Oct)_2_ in the polymer precursor before gelation. The pre‐SiOC was formed after gelation and pyrolyzed to produce the SiOC/Sn nanocomposite product.

In short, the synthesis of the SiOC/Sn nanocomposites was performed in a few steps that include (**Figure** **1**): a hydrosilylation reaction between poly(methylhydrosiloxane) (PMHS) and 200 wt% of divinyl benzene (DVB), yielding a PMHS–DVB polymer precursor with apolar sidechains; the gelation of the polymer precursor with Sn(Oct)_2_ following the formation of the preceramic polymer (pre‐SiOC); and finally, the pyrolysis of pre‐SiOC at 1000 °C. The amount of Sn within the SiOC matrix was controlled by the concentration of Sn(Oct)_2_ in the PMHS–DVB polymer precursor with an upper concentration limit of 60 wt% of Sn(Oct)_2_. At higher concentrations, the solution had a more turbid appearance, which hampered efficient dispersions in the pre‐SiOC. In this work, the SiOC/Sn samples with 10, 25, 40, and 60 wt% of Sn(Oct)_2_ are named SiOC/Sn‐10, SiOC/Sn‐25, SiOC/Sn‐40, and SiOC/Sn‐60, respectively.

**Figure 2:**
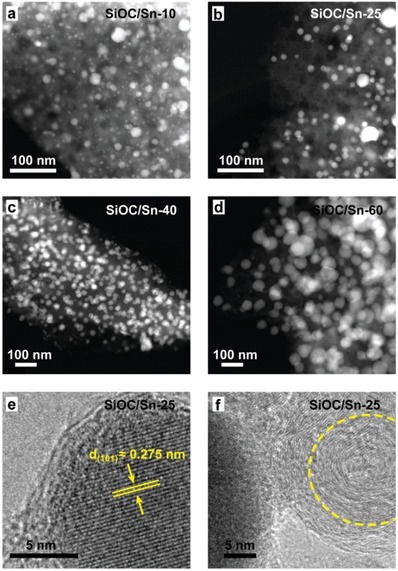
HAADF‐STEM images of the as‐prepared a) SiOC/Sn‐10, b) SiOC/Sn‐25, c) SiOC/Sn‐40 and d) SiOC/Sn‐60 nanocomposites. The Sn NPs (white dots) are homogeneously dispersed within the amorphous SiOC ceramic matrix. e,f) High‐resolution TEM images of SiOC/Sn‐25.

**Figure 3:**
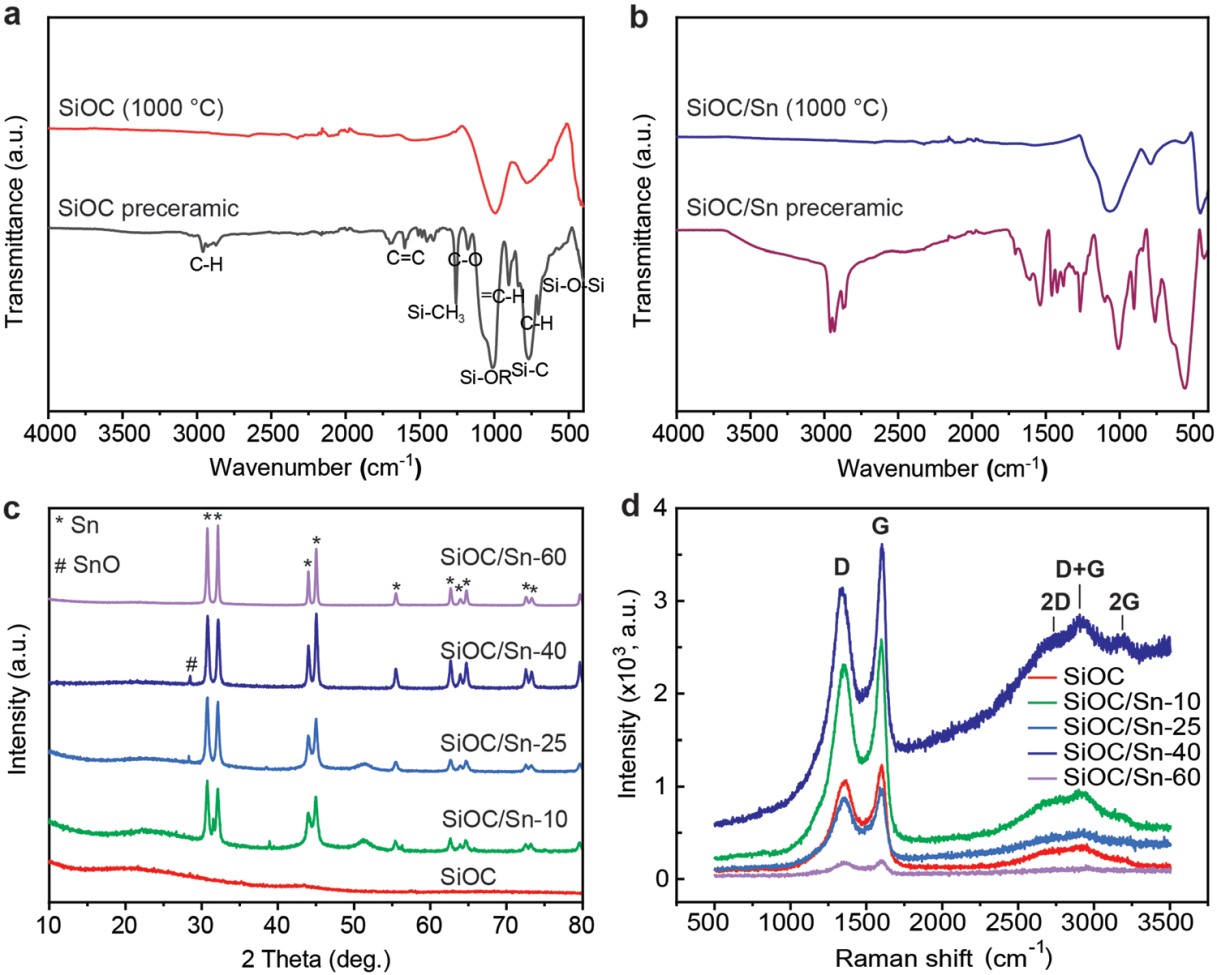
a,b) FTIR spectra of pre‐SiOC, SiOC, pre‐SiOC/Sn, and SiOC/Sn. Pre‐SiOC/Sn and SiOC/Sn samples were synthesized using 40 wt% of Sn(Oct)_2_ in a PMHS–DVB polymer precursor. c) PXRD patterns and d) Raman spectra of SiOC and SiOC/Sn nanocomposites with different Sn contents.

Powder X‐ray diffraction (PXRD), high‐angle annular dark field scanning transmission electron microscopy (HAADF‐STEM), and transmission electron microscopy (TEM) confirmed the formation of uniform and highly crystalline β‐Sn nanoparticles (NPs) in SiOC (tetragonal, space group I4_1_/amd, *a* = *b* = 0.5831, *c* = 3.182 nm, JCPDS No. 00‐004‐0673) with sizes on the order of 5–30 nm (see **Figure** **2**a–d for HAADF‐STEM, Figure 2e and **Figure** **3**c for TEM and PXRD data, respectively). In the SiOC/Sn‐60 sample, however, the size of the Sn NPs increased up to 40 nm. Interestingly, the synthesized SiOC/Sn nanocomposites were also composed of graphited carbon sheets, as highlighted by the yellow circle in the high‐resolution TEM image of SiOC/Sn‐25 (Figure 2f). A broader collection of HAADF‐STEM, TEM, and SEM images can be found in Figure S1 (Supporting Information).

Fourier transform infrared (FTIR) absorption spectra of the pre‐SiOC with and without 40 wt% of Sn(Oct)_2_ and the corresponding pyrolyzed SiOC/Sn and SiOC ceramics are presented in Figure 3, allowing a detailed understanding of the polymer crosslinking process. As follows from Figure 3a, C—H (2900 cm^−1^), C=C (1650 cm^−1^), Si—O—Si (1130 cm^−1^) and Si—CH_3_ (1270 cm^−1^) bonds were observed in the crosslinked pre‐SiOC networks. Importantly, the absence of Si—H bonds at 2100 cm^−1^ in the pre‐SiOC gels demonstrates the effective substitution and subsequent crosslinking between the Si—H bonds from PMHS with DVB. The low intensity of the C=C band at 1650 cm^−1^ can be attributed to an excess of DVB being used in the synthesis. The same structural units, such as C—H, C=C, Si—OR, Si—O—Si, and Si—CH_3_, can be observed in the pre‐SiOC/Sn, with two additional Sn—O and C=O bonds associated with Sn(Oct)_2_. Notably, as observed in the FTIR spectra of pre‐SiOC/Sn, no Sn—O—Si bonds are present, indicating the absence of substitution of Si atoms with the Sn precursor. As shown in Figure 3a,b, both the SiOC and SiOC/Sn ceramics were characterized by the presence of Si—O—Si (1080 cm^−1^) and Si—C (≈800 cm^−1^) bonds, indicating the loss of all organics during pyrolysis.

All pre‐SiOC and pre‐SiOC/Sn powders turned black after pyrolysis at 1000 °C due to the formation of a C_free_ phase. As follows from detailed in situ mass and differential scanning calorimetry (DSC) analysis (Figure S2, Supporting Information), the polymer to ceramic conversion starts at temperatures of 400–600 °C with concomitant losses of organics and other volatile moieties. The latter causes a weight loss in pre‐SiOC in the range of 30–50 wt% during pyrolysis.

The final content of Sn within the SiOC matrices was determined by elemental analysis of Si, O, C, and Sn. The compositions of all the prepared samples, along with the corresponding amount of SiOC and C_free_, are displayed in **Table** **1**. For the pure SiOC ceramic, the SiOC:C_free_ ratio was 55:45 wt%, which is in agreement with previously reported optimized values for C_free_‐rich SiOC. The real Sn content in the SiOC/Sn nanocomposites varied from 6 to 39 wt%, corresponding to 10–60 wt% of Sn(Oct)_2_ in the PMHS–DVB polymer precursor. Interestingly, the C_free_ amount decreased consistently with the increasing amount of Sn(Oct)_2._ This can be explained by the use of C_free_ for the carbothermal reduction of SnO*_x_* (formed upon decomposition of Sn(Oct)_2_) at temperatures above 700 °C.^17^ This assumption can be rationalized by taking a closer look at the Raman spectra of the SiOC/Sn nanocomposites (Figure 3d) showing the well‐known D and G bands at ≈1350 and 1600 cm^−1^. These bands represent the E2g and A1g modes of the vibrations of disordered aromatic carbon and C—C sp^2^ vibrations, respectively.^18^ While the overall intensities show large variations, which cannot be linked directly to the C_free_ content, a general trend in the ratio between the D and the G bands can be observed. The intensity of the D band decreased steadily with increasing Sn content, further demonstrating the effects of carbothermal reduction. Additionally, the second‐order region of the Raman spectrum for SiOC/Sn‐40 shows a high intensity of D+G combination mode at 2940 cm^−1^. The latter can be attributed to the high level of disordering of carbon, which might favorably influence the charge storage capacity of the SiOC matrix.

**Table 1:** Elemental analysis of SiOC and SiOC/Sn nanocomposites and comparison with reported data for SiOC. The explanation of the calculations of C_free_ and SiOC contents is given in the experimental section

Samples	Elemental content [wt%]				Formula (normalized)	SiC*_x_*O_2(1‐_ *_x_*_)_	C_free_	C_Free_	SiOC
	Si	C	O	Sn				[wt%]	
SiOC	29.56	50.04	20.40	–	SiO_1.21_C_3.95_	SiC_0.39_O_1.21_	3.55	44.96	55.04
SiOC/Sn‐10	25.25	42.97	25.60	6.18	SiO_1.77_C_3.97_Sn_0.06_	SiC_0.11_O_1.77_	3.86	41.70	52.12
SiOC/Sn‐25	27.85	36.25	23.40	12.50	SiO_1.47_C_3.04_Sn_0.11_	SiC_0.26_O_1.47_	2.77	32.89	54.61
SiOC/Sn‐40	29.38	13.12	28.90	28.60	SiO_1.72_C_1.04_Sn_0.23_	SiC_0.13_O_1.72_	0.90	11.35	60.05
SiOC/Sn‐60	25.02	10.28	26.20	38.50	SiO_1.83_C_0.96_Sn_0.36_	SiC_0.21_O_1.83_	0.75	8.07	53.43
SiOC 19	30.00	43.00	27.00	–	SiO_1.58_C_3.34_	SiC_0.21_O_1.94_	3.13	37.91	62.09
SiOC^20^	29.24	55.15	14.99	–	SiO_0.90_C_4.40_	SiC_0.55_O_0.90_	3.85	48.53	51.47

### 2.2 Evaluation of the Electrochemical Performance of the SiOC/Sn Nanocomposites

For the electrochemical measurements, the SiOC and SiOC/Sn electrodes were prepared by mixing a powder of SiOC or SiOC/Sn with carbon black, carboxymethyl cellulose (CMC), and water, and the resulting slurries were cast onto a copper foil current collector via doctor‐blading, yielding active material loadings in the range of 2–3 mg cm^−2^. We note that in contrast to the existing literature, where polyvinylidene difluoride (PVDF) is mainly used as a binder, we used a CMC binder, yielding a higher electrochemical performance of SiOC (see Figure S3, Supporting Information for comparison). The choice of CMC was motivated by previous reports showing a vastly improved cycling performance of Si anodes compared to those made with PVDF^21^ due to a stronger bond between the binder and the active material particles. A stronger bond helps retain the electrical contact of the active material with the current collector during the repeated structural changes associated with the lithiation and delithiation of Si and Sn.^22^

**Figure 4:**
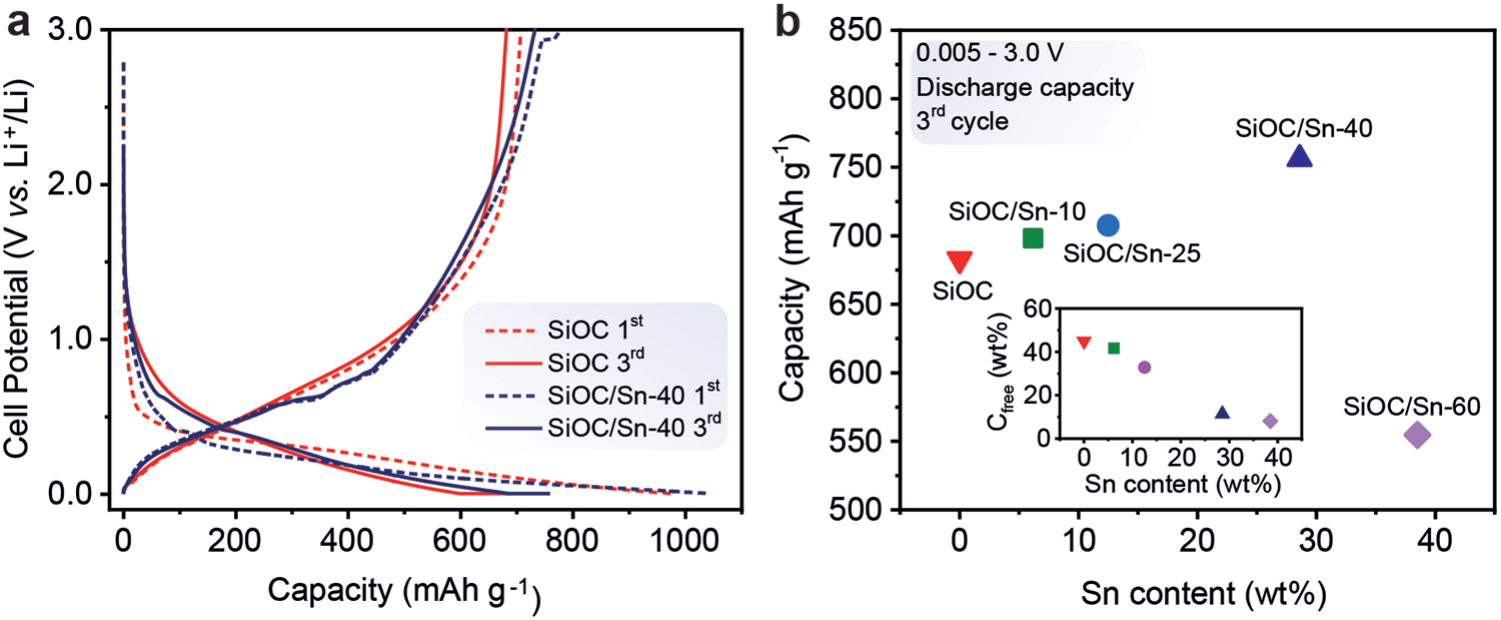
a) Galvanostatic charge–discharge curves of SiOC and SiOC/Sn‐40 during the first and third cycles at current densities of 18.6 and 124 mA g^−1^ between 0.005 and 3.0 V versus Li^+^/Li. b) Corresponding third‐cycle discharge capacity versus Sn content (inset: the dependence of the C_free_ content vs Sn content).

**Figure** **4**a shows the voltage profiles of electrodes comprising SiOC and SiOC/Sn during the first cycle at a current density of 18.6 mA g^−1^ using a constant current‐constant voltage (CCCV) protocol (between 5 mV and 3.0 V vs Li^+^/Li), which applies a constant voltage step of 5 mV. The shape of the voltage profiles of the pure SiOC electrodes was rather smooth, suggesting slow and gradual lithiation of SiOC. Such behavior with stretched‐out features in voltage profiles is rather typical for SiOC, which features different types of active sites for lithium storage, including 1) storage in the interstitial spaces and edges of graphene within C_free_; 2) capacitive storage in the micropores of SiOC; and 3) direct or indirect storage within the amorphous Si—O—C network (see Stabler et al. for an overview of possible Li‐ion storage mechanisms in SiOC).[qv: 12c] In contrast, the electrochemical performance of SiOC/Sn resulted in blurred yet resolvable plateaus associated with the formation of Sn–Li alloys. In the prior work on Sn and other conversion materials, the plateaus in the voltage profiles are often obscured by the very small crystallite size of Sn because the electrochemical reactions at the nanoscale level are known to occur within broader voltage intervals and are affected by surface phenomena. The first complete cycle was characterized by an irreversible capacity loss of 27%, which can be attributed to the formation of the SEI as well as irreversible bonding of Li ions to oxygen sites in tetrahedral SiOC units at the interface with C_free_.^23^ Similar initial coulombic efficiencies for the SiOC and SiOC/Sn electrodes (73% and 74%, respectively) indicate that the irreversibility is not enhanced significantly by the presence of Sn in the matrix. From the third cycle onward, the reversibility improved dramatically, and the capacity becomes stable at a level of ≈683 mAh g^−1^ for SiOC and 756 mAh g^−1^ for SiOC/Sn, along with high coulombic efficiencies of >99% and 97% (>99% from the ninth cycle on), respectively (Figure 4a). Importantly, a comparison of the electrochemical performances of SiOC and SiOC/Sn after the third cycle revealed a steady increase in the reversible capacity from 683 to 756 mAh g^−1^ with increasing Sn content up to 40 wt% (Figure 4b and Figure S4, Supporting Information). The latter can be explained by the higher capacity of Sn (994 mAh g^−1^) in comparison with that of SiOC; the latter is in the range of 600–700 mAh g^−1^ at a well‐optimized C_free_:SiOC ratio. As mentioned above, upon increasing the Sn content, the contribution of C_free_ drastically decreased from 50 to 10 wt%, which in combination with Sn is sufficient to provide an electronic pathway through a nonconductive SiOC matrix (Figure 4b). However, upon increasing the Sn concentration up to 60 wt%, the C_free_ content decreased to approximately 8 wt%. No linearity between the capacity and the segregated carbon content has been found previously.^24^ However, the reduction of C_free_ hampers electronic transport,^25^ which, along with the very close proximity of Sn NPs, explains the large and rapid capacity decrease from 555 mAh g^−1^ (third cycle) to 244 mAh g^−1^ (tenth cycle) for the SiOC/Sn‐60 sample (Figure S4, Supporting Information).

**Figure 5:**
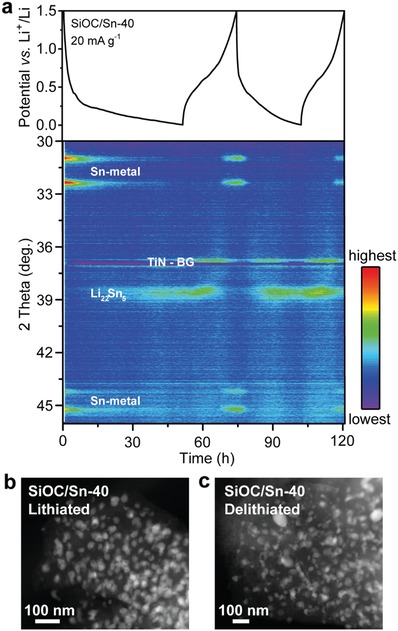
a) In situ XRD patterns of SiOC/Sn‐40 during electrochemical cycling versus Li metal at a current density of 20 mA g^−1^. The active material loading is ≈3 mg cm^−2^. The XRD peak at a 2θ of 37° is attributed to the titanium nitride/polyimide current collector labeled TiN‐BG. b,c) HAADF‐STEM images of SiOC/Sn‐40 after discharge (lithiation) and charge (delithiation).

To trace the evolution of crystalline Sn‐containing phases in an amorphous SiOC matrix, in situ XRD measurements were performed (**Figure** **5**a). During discharge, a steady decrease in the intensity of the Sn diffraction peaks at 2θ values of 31° and 32.5° and the concomitant appearance of peak at 38.5–39°, which is associated with the formation of a Li_22_Sn_5_ alloy, indicate the lithiation of the Sn NPs. Upon charging, the Li_22_Sn_5_ and Sn peaks vanish and then reappear, indicating delithiation and the formation of metallic Sn. These results are in agreement with previous in situ XRD studies showing that lithiation of Sn results in the formation of a Li_22_Sn_5_ alloy upon full lithiation.^26^ Importantly, as observed by TEM, no evidence of significant Sn NP aggregation was found upon lithiation and delithiation of SiOC/Sn (see Figure 5b,c).

**Figure 6:**
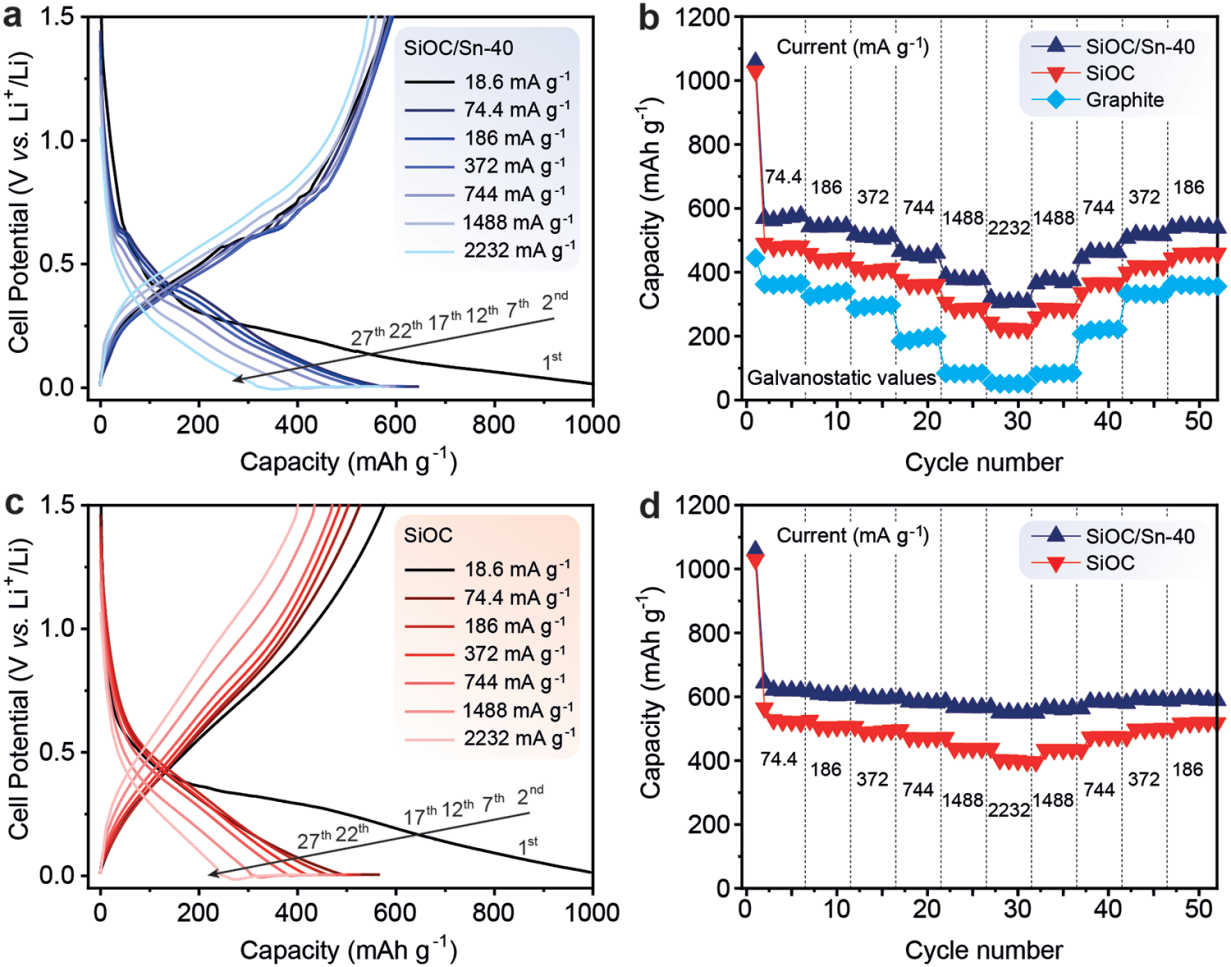
a) Galvanostatic charge–discharge curves of SiOC/Sn‐40. Discharge capacities of SiOC/Sn‐40, SiOC, and graphite b) without and d) with a constant voltage step contribution measured at different current densities ranging from 18.6 to 2232 mA g^−1^. c) Galvanostatic charge–discharge curves of SiOC.

Next, we tested the rate‐capability performance of SiOC and SiOC/Sn‐40 using an upper voltage limit of 1.5 V versus Li^+^/Li. The voltage range of 5 mV–1.5 V is more relevant for practical applications than the range of 5 mV–3 V, which is commonplace in the literature, including reports on SiOC.^11^, ^14^, ^16^, ^19^, ^20^, ^27^ **Figure** **6**a,c shows the voltage profiles of the nanocomposite SiOC/Sn‐40 and SiOC at different currents, ranging from 74.4 to 2232 mA g^−1^ and yielding capacities ranging from 644 to 553 mAh g^−1^ for SiOC/Sn‐40. The corresponding capacities without (galvanostatic) and with a constant voltage step contribution are presented in Figure 6b,d, respectively. As shown in Figure 6b, the galvanostatic capacity of SiOC/Sn‐40 was as high as 456 mAh g^−1^ at a current density of 744 mA g^−1^ and 92 mAh g^−1^ higher than that of pure SiOC. In addition, these results represent a 3.5‐fold increase in the rate capability compared to the previously reported values for SiOC/Sn anode materials,^15^ indicating higher electronic conductivity and faster charge‐transfer kinetics in the SiOC/Sn‐40 anode. Even at a current density of 2232 mA g^−1^, an impressive galvanostatic capacity of 309 mAh g^−1^ can be reached. Notably, when the current density returned to 186 mA g^−1^ from a high current density of 2232 mA g^−1^, nearly complete capacity recovery was achieved. Importantly, these results compare favorably with the state‐of‐the‐art Sn‐carbon anode materials (see Table S1, Supporting Information).

To present a fair comparison of the electrochemical performance of SiOC/Sn‐40 with graphite, we prepared and tested graphite electrodes with comparable active material loadings of 2.3 mg cm^−2^ and electrode compositions. The graphite electrodes demonstrate high galvanostatic capacities of 362 mAh g^−1^ at 74.4 mA g^−1^ (C/5 rate, Figure 6b). At high lithiation rates of 2232 mA g^−1^ (6C), however, the capacity typically decreased to 44–51 mAh g^−1^; this result is commensurate with the state‐of‐the‐art reports on graphite electrodes with similar area capacities.^28^ A detailed experimental comparison of the gravimetric capacities of the SiOC/Sn‐40, SiOC, and graphite electrodes at different current densities can be found in Figure S5 (Supporting Information). Next, we analyzed the theoretical energy densities of full cells comprising a LiFePO_4_ cathode versus either graphite, SiOC, or SiOC/Sn‐40 anode. We estimated that SiOC/Sn‐40 at a 186 mA g^−1^ delivers an energy density of ≈353 Wh kg^−1^, which is on par with the values obtained with a graphite anode, offering no apparent advantage (see **Table** **2**). The low energy density of SiOC and SiOC/Sn‐40 is primarily caused by the high delithiation potential of SiOC and SiOC/Sn‐40, namely, 0.6–0.7 V, which is considerably higher than 0.2–0.46 V versus Li^+^/Li for graphite. However, at higher rates, such as 2232 mA g^−1^, the value is 300 Wh kg^−1^ for SiOC/Sn‐40 but 114 Wh kg^−1^ for graphite. These results highlight the primary advantage of SiOC/Sn over graphite‐based electrodes, which is its far greater utility for high power energy storage applications.

**Table 2:** Comparison of the energy density of SiOC/Sn‐40 with SiOC and graphite in a full cell. The values displayed are taken versus an ideal LiFePO_4_ counter electrode without overpotential (average voltage of 3.4 V, the capacity of 165 mAh g^−1^).[qv: 1d] All capacities are taken from Figure 6b and are based on galvanostatic values from a two‐electrode half‐cell

Material	Average delithiation voltage [V] at 186–2232 mA g^−1^	Avg. voltage in a full cell (vs LiFePO_4_)	Energy density [Wh kg^−1^] at 186 mA g^−1^	Energy density [Wh kg^−1^] at 744 mA g^−1^	Energy density [Wh kg^−1^] at 2232 mA g^−1^
SiOC	0.71–0.83	2.69–2.57	324	302	246
SiOC/Sn‐40	0.61–0.70	2.79–2.70	353	334	300
Graphite SLP‐50	0.21–0.46	3.19–2.93	349	271	114

**Figure 7:**
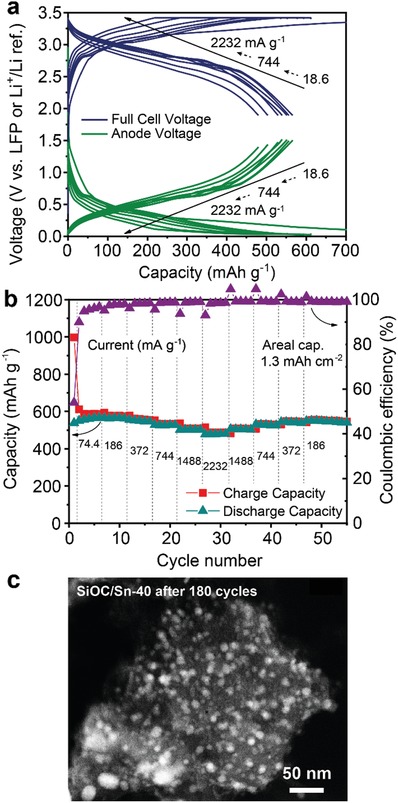
Electrochemical performance of anode‐limited Li‐ion full cell comprising a SiOC/Sn‐40 anode and LiFePO_4_ cathode in a three‐electrode configuration. a) Galvanostatic charge/discharge curves of the SiOC/Sn‐40/LiFePO_4_ full cell and SiOC/Sn‐40 versus Li^+^/Li. b) Charge and discharge capacities of SiOC/Sn‐40 measured at different current densities. c) HAADF‐STEM micrograph of SiOC/Sn‐40 cycled 180 times in a full cell versus LiFePO_4_.

To assess the practical utility of SiOC/Sn as an anode, anode‐limited full cells using LiFePO_4_ as the cathode were assembled. Specifically, the full cells were composed of a LiFePO_4_ cathode (areal capacity of 3.5 mAh cm^−2^) and a SiOC/Sn‐40 nanocomposite anode (areal capacity of ≈1.3 mAh cm^−2^). The analysis was performed in a three‐electrode cell configuration with ring‐shaped lithium as a reference electrode. Herein, all specific capacities and currents correspond to the mass of SiOC/Sn‐40. As shown in **Figure** **7**, SiOC/Sn‐40 exhibits discharge capacities of 567, 563, 551, 527, 502, and 479 mAh g^−1^ upon cycling at 74.4, 186, 372, 744, 1488, and 2232 mA g^−1^, respectively, showing similar results to those obtained in the half‐cell experiments.

The tested batteries had a capacity retention of 81% after 100 cycles at 186 mA g^−1^ (see Figure S6, Supporting Information). Importantly, the average discharge voltage was stable with increasing discharge rate: 2.76 V at 186 mA g^−1^, 2.73 V at 744 mA g^−1^ and 2.63 V at 2232 mA g^−1^. Neglecting the excess of LiFePO_4_ cathode (≈165 mAh g^−1^)^[1d]^, the charge storage capacity of the cell was estimated as 352 Wh kg^−1^ at 186 mA g^−1^, 343 Wh kg^−1^ at 744 mA g^−1^, and 322 Wh kg^−1^ at 2232 mA g^−1^ with a resulting power density up to 1.5 kW kg^−1^. The HAADF‐STEM images of cycled SiOC/Sn‐40 (Figure 7c and Figure S7, Supporting Information) show only minor particle pulverization or aggregation after 180 cycles in the full‐cell configuration, thus further demonstrating the effectiveness of the SiOC matrix in stabilizing Sn NPs. Lastly, equilibrated full cells comprising SiOC/Sn anode and LiFePO_4_ cathode were assembled (see Figure S8, Supporting Information), and showed slow capacity fading. The latter can be attributed to the formation of SEI on SiOC/Sn anode causing significant lithium losses within the initial 10 cycles. These results suggest that future work should be focused on the development of effective chemical prelithiation solutions of SiOC toward compensation of lithium consumption caused by SEI formation.

## 3 Conclusions

In summary, we have reported a facile synthesis of a SiOC/Sn nanocomposite by the pyrolysis of a preceramic single‐source precursor. The latter was devised from Sn(Oct)_2_ and poly(methylhydrosiloxane) grafted with apolar divinyl benzene side‐chains. This molecular engineering of the precursors allows for intimate blending of Sn(Oct)_2_ with the polymeric backbone and yields, after thermal decomposition, a homogeneous distribution of Sn nanoparticles (5–30 nm) within the SiOC matrix. Neat SiOC ceramics and SiOC/Sn nanocomposites with different Sn contents of 10, 25, 40, and 60 wt% were thoroughly tested electrochemically. SiOC/Sn‐40 demonstrated the highest Li‐ion storage capacity of 756 mAh g^−1^ at a current density of 124 mA g^−1^ (≈C/3 rate for the graphite anode). The lithiation/delithiation mechanism of SiOC/Sn‐40 was corroborated by in situ XRD and ex situ TEM data indicating the reversible formation of the Li_22_Sn_5_ alloy and the absence of significant Sn aggregation within the amorphous SiOC matrix upon lithiation/delithiation. The key attribute of the SiOC/Sn‐40 composite is its high rate capability. For instance, a high galvanostatic capacity of 309 mAh g^‐1^ was obtained at a current density of 2232 mA g^‐1^ (≈6 C for the graphite anode) with a high mass loading of 2.64 mg cm^−2^ (corresponding to an areal capacity of ≈1.3 mAh cm^−2^). For comparison, standard graphite anodes with a comparable areal capacity and similar electrode composition yielded rather inferior results—a capacity of 44 to 51 mAh g^−1^ at a current density of 2232 mA g^‐1^. In light of these results, SiOC/Sn‐40, therefore, might be considered a compelling anode material in Li‐ion batteries for high‐power energy storage applications. Future work should focus on studying and optimizing long‐term cycling stability. Another promising avenue is to extend the SiOC matrix concept to antimony and hard carbon materials with prospects for high‐rate and high‐capacity Na‐ion storage.

## 4 Experimental Section

*Chemicals for the Synthesis of SiOC and SiOC/Sn*: Polyhydromethyl siloxane (MW ≈1900, PMHS, Aldrich), divinyl benzene (DVB, technical grade, 80%, Aldrich), tin 2‐ethyl‐hexanoate (Sn(Oct)_2_, 92.5‐100.0%, Sigma‐Aldrich), and platinum Karstedt's catalyst (platinum(0)‐1,3‐divinyl‐1,1,3,3‐tetramethyldisiloxane complex solution in xylene, Pt ≈2%, Sigma‐Aldrich) were used as received.

*Battery Components*: Carboxymethyl cellulose (CMC, SUNROSE MAC 500LC, Nippon Paper Group), poly(vinylidene fluoride) (PVDF, *M*_w_ ≈534 000, Sigma‐Aldrich), carbon black (CB, Super P, TIMCAL, Switzerland), ethylene carbonate (EC, battery grade, BASF), LiPF_6_ (battery grade, Novolyte Technologies), graphite (SLP50, TIMCAL), dimethyl carbonate (DMC, battery grade, BASF), and fluoroethylene carbonate (FEC, >98%, TCI Chemicals) were used as received. Lithium iron phosphate cathodes were purchased from Custom Cells (areal capacity of 3.47 mAh cm^−2^, Batch Nr. I0S0F).

*Synthesis of SiOC*: In a typical synthesis of SiOC ceramics, PMHS (1 g), DVB (2 g), and platinum Karstedt's catalyst (5 µL) were combined into a 25 mL flask, and the mixture was stirred at room temperature for 30 min. The obtained preceramic polymer gel was then aged in a drying oven at 80 °C under a normal atmosphere (air) for 48 h, followed by pyrolysis at 1000 °C. The latter was carried out in an alumina tubular furnace under a controlled argon atmosphere (Carbolite STF 16/450, Germany) with heating and cooling rates of 60 °C h^‐1^ and a holding time of 1 h at the dwell temperature.

*Synthesis of SiOC/Sn Nanocomposites*: In a typical synthesis of SiOC/Sn, PMHS (1 g), DVB (2 g), and a platinum Karstedt's catalyst (5 µL) were combined into a 25 mL flask, and the mixture was stirred at room temperature for 30 min. Sn(Oct)_2_ (0.35, 1, 2, or 4.5 g, corresponding to 10, 25, 40, and 60 wt%, respectively) was added, and the reaction mixture was stirred at RT for 30 min; gelation was observed within 3 h. The obtained preceramic polymer gel was aged via heat treatment in a drying oven at 80 °C for 48 h. After heat treatment, the preceramic SiOC/Sn powder was pyrolyzed in a manner analogous to that of the pure SiOC ceramics.

*Characterization*: Attenuated total reflectance (ATR) FTIR spectra were recorded using a Bruker Tensor 27 spectrometer (Bruker, USA) equipped with a Golden Gate ATR crystal. Raman spectra of the samples were recorded by a confocal Raman spectrometer (Renishaw, UK) using a laser beam of 488 nm. The contents of carbon, oxygen and tin in the SiOC and SiOC/Sn samples were determined by elemental analysis with Mikroanalytisches Labor Pascher (Remagen‐Bandorf, Germany). The content of silicon was estimated as the difference between the total amount of all elements (100 wt%) and the sum of O, C, and Sn contents (in wt%). The SiC*_x_*O_2(1‐_ *_x_*_)_ and C_free_ = C_total_ – C*_x_* formulas were applied for calculations of the ratio between SiOC and C_free_ based on the total amount of Si, O, and C, which was determined from the elemental analysis.

*Electrode Fabrication*: SiOC, SiOC/Sn, or graphite SLP50 (680 mg, 85%) and carbon black (60 mg, 7.5%) were ball‐milled in a planetary ball‐mill at 350 rpm for 30 min. CMC (60 mg in water (2.4 mL), 7.5%) or PVDF (60 mg in NMP (2.4 mL), 7.5%) was added to the dry mass, and the mixture was ball‐milled in a planetary ball‐mill at 350 rpm for 1 h. The obtained slurry was coated onto Cu‐foil using a doctor‐blading technique and then dried overnight in a vacuum oven at 120 °C (2 × 10^−2^ mbar). Electrode active material loadings were in the range of 2–3 mg cm^−2^.

*Cell Assembly and Testing*: Stainless‐steel coin‐type cells (CR2025) were assembled in a glovebox under an inert Ar atmosphere (<0.1 ppm H_2_O/O_2_) with a glass microfiber separator and 130 µL of 1 m LiPF_6_ in EC:DMC (1:1 by weight) with 3 wt% FEC. For the three‐electrode cell, an EL‐CELL PAT‐Cell (A 0001) with a ring‐shaped Li‐reference electrode and 80 µL of electrolyte was used. The diameter of the electrodes was 18 mm. The coin cells were electrochemically cycled after a waiting time of 2 h using a multichannel workstation (Astrol BAT‐Flex). Full cell testing in a three‐electrode configuration was performed using a separate multichannel workstation (MPG‐2, Bio‐Logic SAS). The purpose of using three‐electrode cell was to separate the voltage contributions of SiOC/Sn anode and LiFePO_4_ cathode from the total cell voltage. To determine the gravimetric capacities and specific energy densities, the full mass of the SiOC and SiOC/Sn composites (85 wt% of the total electrode material) was used.

*In Situ XRD Measurements*: Modified stainless (CR2025) coin‐type cells covered with TiN‐coated Kapton foil (see Rhodes et al.^29^ and Wang et al.^30^ for fabrication details) were used for in situ XRD measurements. SiOC/Sn‐40 (3.42 mg active material) was coated onto the TiN side. The electrode was dried under vacuum at 80 °C for 12 h. The rest of the battery was assembled in a glovebox under an inert Ar atmosphere (<0.1 ppm H_2_O/O_2_) with a glass microfiber separator and a Li metal reference electrode. The XRD measurements were conducted on a Bruker AXS D8 Advance X‐ray diffractometer in reflection mode combined with a multichannel workstation (MPG‐2, Bio‐Logic SAS). The cell was cycled in the voltage range 0.005–1.5 V at 20 mA g^−1^ and a 2θ range of 15° to 60° every 36 min. A background with a coin cell and TiN Kapton‐foil was recorded and subtracted.

## Conflict of Interest

The authors declare no conflict of interest.

## Supporting information

Supplementary
