# Supplementary material for: Silicon Oxycarbide—Tin Nanocomposite as a High‐Power‐Density Anode for Li‐Ion Batteries
Source: Adv Sci (Weinh). 2019 Jul 28;6(19):1901220. doi: 10.1002/advs.201901220 (PMC6774025; doi:10.1002/advs.201901220)
Supplement: Supplementary file 1 — Supplementary [file ADVS-6-1901220-s001.pdf]

Copyright WILEY-VCH Verlag GmbH & Co. KGaA, 69469 Weinheim, Germany, 2019.

ADVANCED SCIENCE
Open Access

Supporting Information

for *Adv. Sci.,* DOI: 10.1002/advs.201901220

Silicon Oxycarbide—Tin Nanocomposite as a High-Power-Density Anode for Li-Ion Batteries

Romain J.-C. Dubey, Pradeep Vallachira Warriam Sasikumar, Frank Krumeich, Gurdial Blugan, Jakob Kuebler, Kostiantyn V. Kravchyk, Thomas Graule, and Maksym V. Kovalenko**

Copyright WILEY-VCH Verlag GmbH & Co. KGaA, 69469 Weinheim, Germany, 2019.

Supporting Information

Silicon Oxycarbide - Tin Nanocomposite as a High-Power-Density Anode for Li-Ion Batteries

Romain J.-C. Dubey, Pradeep Vallachira Warriam Sasikumar, Frank Krumeich, Gurdial Blugan, Jakob Kübler, Kostiantyn V. Kravchyk, Thomas Graule and Maksym V. Kovalenko**

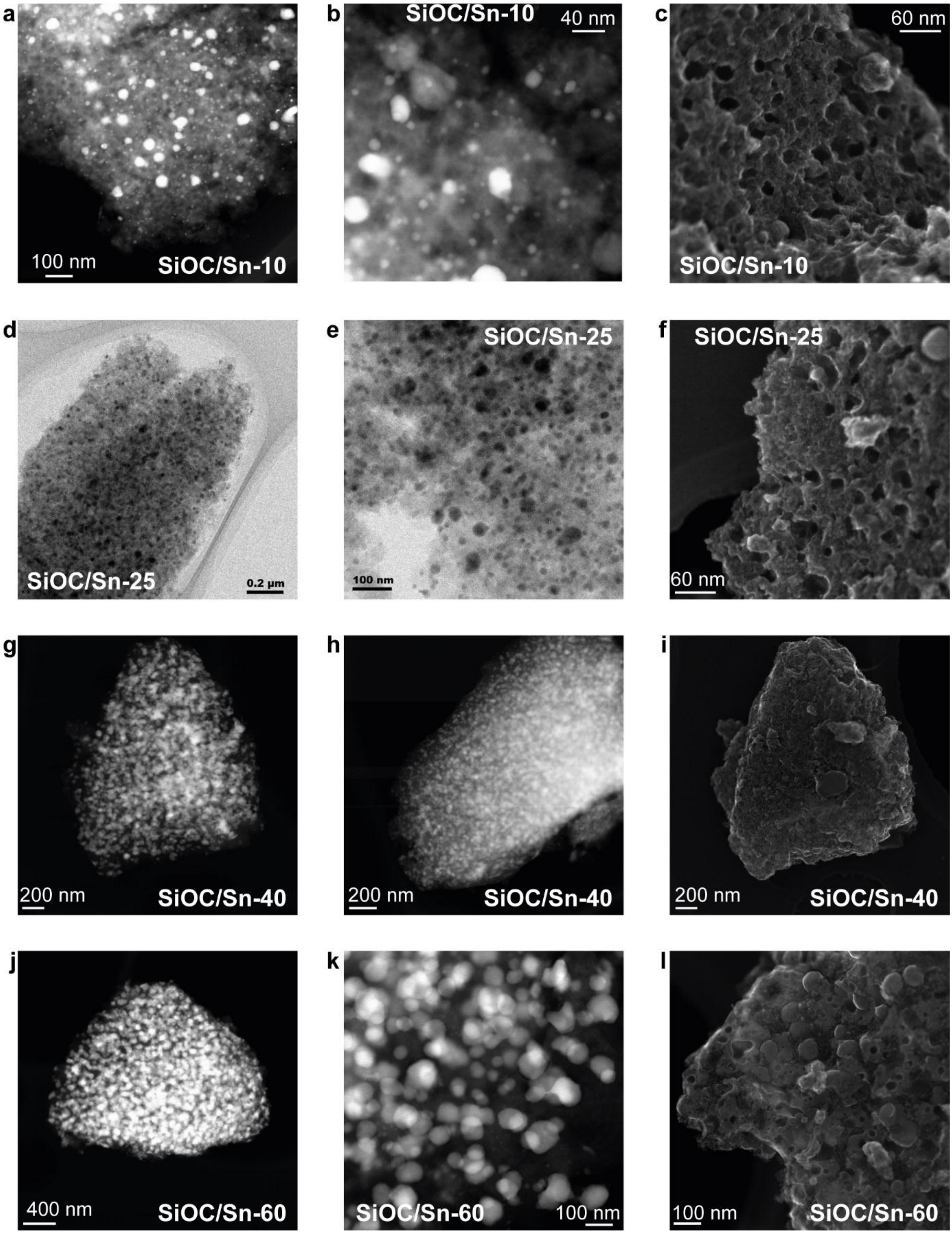

Figure S1. HAADF-STEM, TEM and SEM micrographs of SiOC/Sn-10, SiOC/Sn-25, SiOC/Sn-40 and SiOC/Sn-60 nanocomposites.

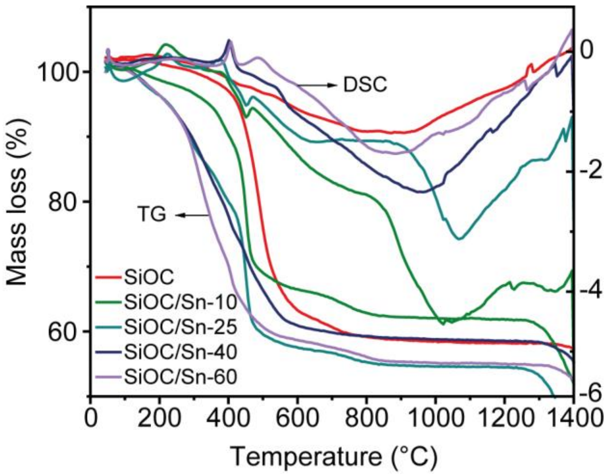

Figure S2. *In-situ* thermogravimetric analysis and differential scanning calorimetry data of preceramic polymers corresponding to SiOC, SiOC/Sn-10, SiOC/Sn-25, SiOC/Sn-40 and SiOC/Sn-60 samples.

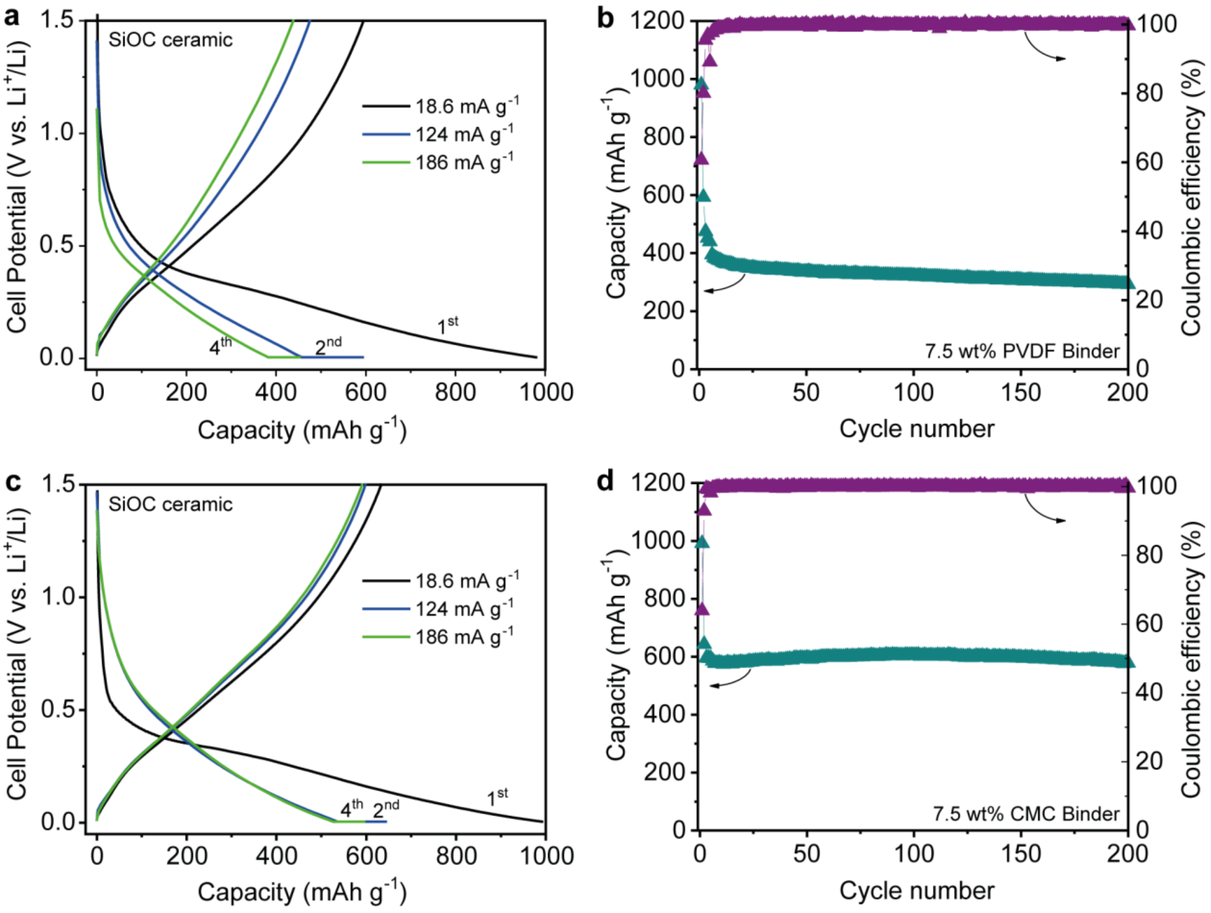

Figure S3. Galvanostatic charge–discharge voltage curves and cycling stability of Li-ion half-cells employing SiOC anodes prepared using PVDF (a, b) and CMC (c, d) binders, accordingly.

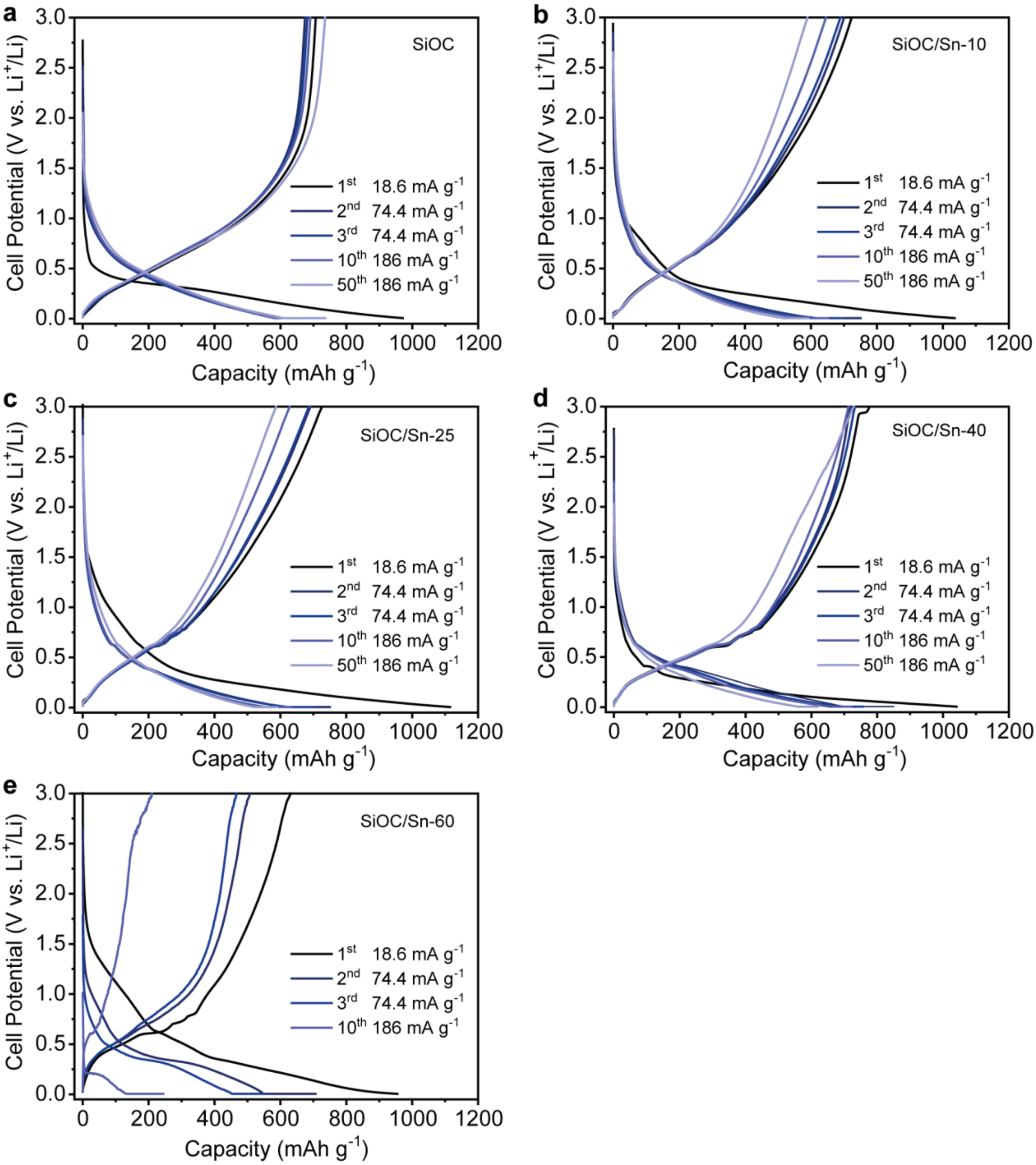

Figure S4. (a-e) Galvanostatic charge-discharge curves of SiOC (a), SiOC/Sn-10 (b), SiOC/Sn-25 (c), SiOC/Sn-40 (d) and SiOC/Sn-60 (e) measured during 1st, 2nd, 3rd, 10th and 50th cycles in the voltage window of 0.005 – 3.0 V.

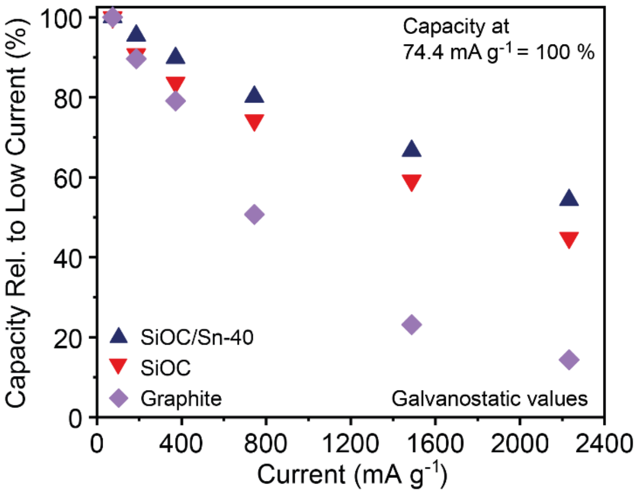

Figure S5. Comparison of the relative galvanostatic capacities of SiOC, SiOC/Sn-40, and graphite anodes in the voltage range of 0.005 – 1.5 V at different current densities.

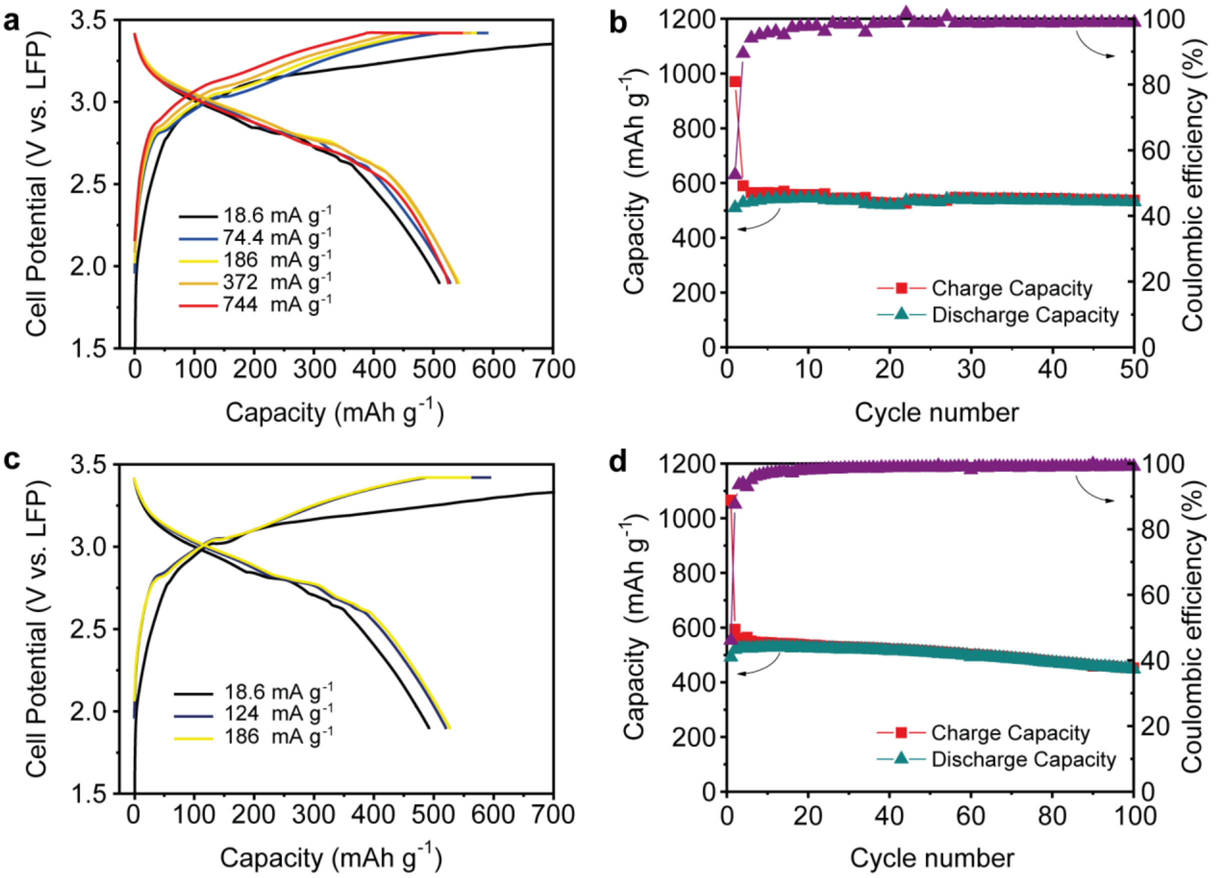

Figure S6. Electrochemical performance of a Li-ion full-cell comprising SiOC/Sn-40 anode and $LiFePO_4$ cathode. (a, b) Galvanostatic discharge/charge curves (a) and discharge/charge capacities (b) of SiOC/Sn-40 anode measured *vs*. LFP cathode at different current densities. (c, d) Galvanostatic discharge/charge curves and discharge/charge capacities (b) of SiOC/Sn-40 anode measured *vs*. $LiFePO_4$ cathode for 100 cycles at current density of 186 mA g^{-1} (1st cycle: at 18.6 mA g^{-1}; 2nd and 3rd cycle at 124 mA g^{-1}, from the 4th cycle and forth at 186 mA g^{-1}).

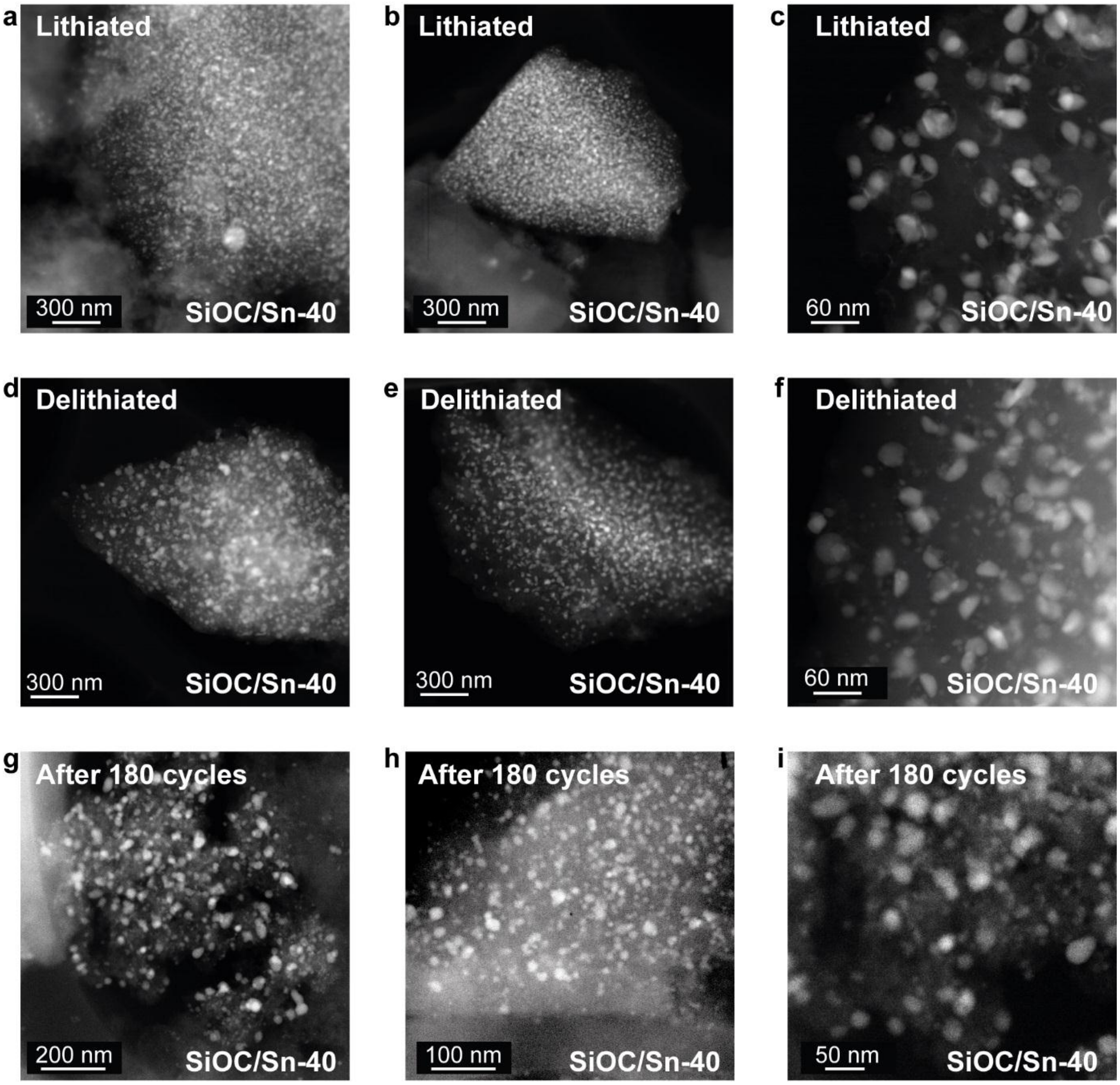

Figure S7. Low-resolution and high-resolution HAADF-STEM micrographs of SiOC/Sn-40 after lithiation (a, b, c), after delithiation (d, e, f) and after 180 cycles in a full cell *vs.* $LiFePO_4$.

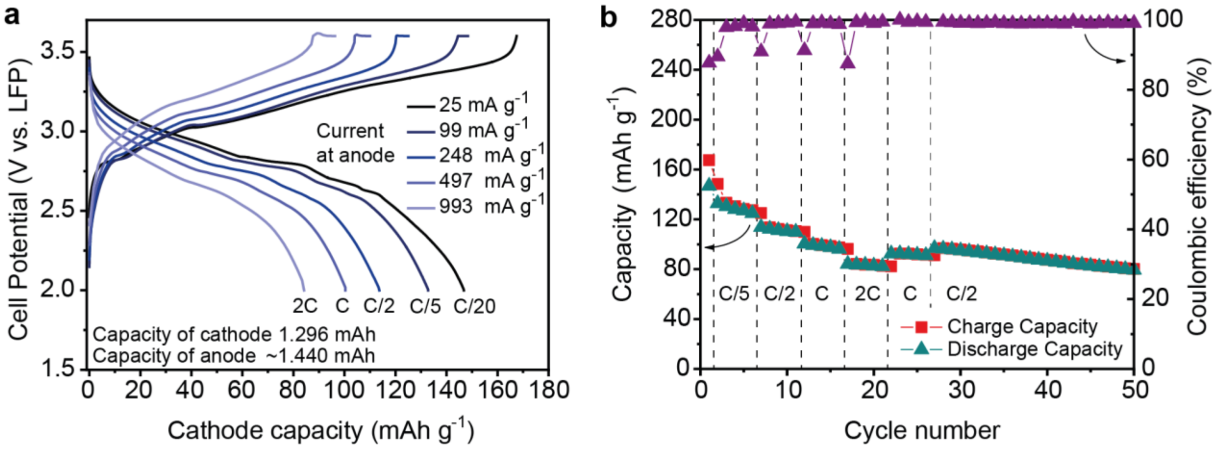

Figure S8. Electrochemical performance of equilibrated Li-ion full cell comprising an SiOC/Sn-40 anode (10 % oversized anode) and a $LiFePO_4$ cathode. (a) Charge/discharge curves and (b) corresponding charge/discharge capacities of full cell measured at current densities from 25 mA g^{-1} to 993 mA g^{-1}. 1C = 1.296 mA. The full cell was assembled after a single lithiation/delithiation cycle of SiOC/Sn-40 anode in the voltage range of 5 mV – 1.5 V at current density of 18.6 mA g^{-1}.

Table S1. Comparative overview of high-performance Sn-based anodes by active material loadings, highest rates and capacities, estimated capacities in a practical voltage range and corresponding observations concerning the practicability of the systems.

**Material**	**Loading (mg cm^{-2})**	**Highest rate (mA g^{-1})**	**Capacity at highest rate (mAh g^{-1})***	**Capacity up to 1.5 V (mAh g^{-1})***	**Observation**	**Reference**
Sn/SiOC composite	4.5	744	133	Unknown	5 mV – 2.5 V	S1
Sn/SiOC nanosheets	7	1400	<100	~700 (35 mA g^{-1})	Low electrode density	S2
Sn filled CNTS	-	2000	429	~875 (100 mA g^{-1})	Flame aerosol deposition and chemical vapor deposition	S3
Nano Sn/C	1	15120	~100 – 250	~470 (200 mA g^{-1})	70 wt% active material Requires spray pyrolysis	S4
Porous carbon/Sn	-	4000	180	~400 (200 mA g^{-1})	70 wt% active material Convenient synthesis	S5
Sn-C hybrid nanocomposite	1.9	744	300	~500 (74.4 mA g^{-1})	70 wt% active material	S6
Porous carbon/Sn	0.5	1000	300	574 (20 mA g^{-1})	Stable for 15 cycles	S7
Sn Graphene composite	-	10000	270	~770 (200 mA g^{-1})	Requires chemical vapor deposition 80 wt% active material	S8
Sn/N-doped carbon microcage	0.8	10000	323	~660 (200 mA g^{-1})	Spray drying - pyrolysis 80 wt% active material	S9
Sn@C nanocomposites	1	16 000	205	~500 (200 mA g^{-1})	70 wt% active material Requires spray pyrolysis	S10
Porous Sn@C nanocomposite	-	4000	300	~850 (200 mA g^{-1})	80 wt% active material Convenient synthesis	S11
Sn NPs encapsulated in 3D nanoporous carbon	-	5000	300	~500 (200 mA g^{-1})	80 wt% active material Convenient synthesis	S12
Sn@C biotemplated	-	2000	155	~400 (50 mA g^{-1})	70 wt% active material	S13
In situ ultrasmall tin particles on crumpled N-doped graphene	1.06	5000	330	~750 (100 mA g^{-1})	75 wt% active material	S14

SiOC/Sn composite	2.64	Half cell<br>2232<br>Full cell<br>2232	With CV step<br>553<br>Galvanostatic<br>309<br>Galvanostatic<br>479	Galvanostatic<br>549 (186 mA g^{-1})	85 wt% active material<br>Convenient synthesis	**This work**

***** These values have been estimated by eye from capacity plots or existing voltage profiles having a broader voltage range and solely serve the purpose of having a starting point for a fair comparison. The observations represent an opinion of the authors and do not constitute a criticism of the published work.

References

[S1] Kaspar et al., *Adv. Funct. Mater.* **2014**, 24, 4097-4104.

[S2] Tolosa et al., *Sustainable Energy Fuels* **2018**, 2, 215–228.

[S3] Hou et al., *ACS Appl. Mater. Interfaces* **2013**, 5, 6672−6677.

[S4] Xu et al., *Nano Lett.* **2013**, 13, 470−474.

[S5] Xu et al., *J. Mater. Chem.* **2012**, 22, 9562.

[S6] Liu et al., *Chem. Commun.* **2017**, 53, 13125-13128.

[S7] Xu et al., *Adv. Energy Mater.* **2012**, 3, 128-133.

[S8] Qin et al., *ACS Nano* **2014**, 82,1728-1738.

[S9] Ying et al., *J. Mater. Chem. A* **2017**, 5, 8334-8342.

[S10] Zhang et al., *Nanoscale* **2014**, 6, 2827-2832.

[S11] Zhang et al., *Nanoscale* **2015**, 7, 11940-11944.

[S12] Guo et al., *ACS Appl. Mater. Interfaces* **2017**, 9, 17172-17177.

[S13] Tao et al., *ACS Appl. Mater. Interfaces* **2014**, 6, 3696-3702.

[S14] Liu et al., *J. Power Sources* **2016**, 328, 482-491.
